# Study protocol: using the Q-STEPS to assess and improve the quality of physical activity programmes for the elderly

**DOI:** 10.1186/1756-0500-5-171

**Published:** 2012-07-09

**Authors:** Ana I Marques, Maria J Rosa, Marlene Amorim, Pedro Soares, António Oliveira-Tavares, Rute Santos, Jorge Mota, Joana Carvalho

**Affiliations:** 1 Research Centre in Physical Activity, Health and Leisure - Faculty of Sports, Porto University, Rua Dr. Plácido Costa, 91-4200 450, Porto, Portugal; 2 Department of Economics, Management and Industrial Engineering, University of Aveiro, Campus Universitário de Santiago, 3810-193, Aveiro, Portugal; 3 José Estêvão High School, Avenida 25 de Abril, 3811-901, Aveiro, Portugal

**Keywords:** Q-STEPS, Feasibility, Physical activity programmes, Elderly, Evaluation, Quality

## Abstract

**Background:**

Aging is one of the most important and obvious phenomenon observed in our society. In the past years, there has been a growing concern in designing physical activity (PA) programmes for elderly people, because evidence suggests that such health promotion interventions may reduce the deleterious effects of the ageing process. Accordingly, a growing body of literature points to the importance of a sound approach to planning and evaluation in order to improve the quality of PA programmes. However, while numerous PA programmes have been designed for the elderly in recent years, their evaluation has been scarce. Quality management processes and tools provide a practical way for organisations to assess, identify and shed light on the areas requiring improvement. The Quality Self-assessment Tool for Exercise Programmes for Seniors (Q-STEPS) seems to provide a framework tailored to evaluate PA programmes for the elderly.

**Findings:**

The primary purpose of this study is 1) to determine feasibility, acceptability and usability of the Q-STEPS. Secondary purposes of the study are: 2) to examine the quality of the PA programmes for elderly people developed by the Portuguese Local Administration over a three-year period of self-assessments in terms of: a) Enabler domains (Leadership, Policy and Strategy, People, Partnership and Resources, Processes); b) Result domains (Customer Results, People Results, Society Results and Key Performance Results); 3) to estimate the association between the use of Q-STEPS and some indicators relating to the elderly participants, during the three self-assessments, such as: attendance rates, physical fitness, health-related quality of life and the elderly’s perceived quality of the programme. The study will be conducted in PA programmes for elderly adults from mainland Portuguese municipalities over a three-year period. The project will adopt a participative quality improvement approach that features annual learning cycles of: 1) self-assessment with the Q-STEPS; 2) feedback to and interpretation of results involving programme’s staff; 3) action planning to achieve system changes; 4) implementation of strategies for change; and 5) review process through further self-assessment. The study will collect a range of process and outcome data that will be used to achieve the research aims.

**Discussion:**

It is our understanding that the results of the Q-STEPS study will contribute directly to the evidence based on effectiveness of continuous quality improvement approaches, in order to improve customer satisfaction and adherence to PA programmes targeting the ageing population. This comprehensive evaluation will also add significant new knowledge regarding the characteristics associated with a sustainable public service.

## Findings

### Background

According to the EUROSTAT, Portugal is one of the ten most aged countries of Europe [1]. The most important issue related to demographic ageing deals with its implications for the well-being of the elderly, such as access to appropriate health-care services. In developed countries, some degree of progress has been made to achieve this objective, all the more so as ageing is the most important contributor to the increase in health care costs [2]. In fact, biopsychosocial changes arising from the ageing process can negatively affect the quality of life of the elderly by limiting their ability to carry out everyday activities and exposing them to a greater vulnerability of health problems [3]. Evidence provided by several studies highlights that physical activity (PA) can play a major role in global health promotion [4,5], in large part by epidemiological evidence of the positive effect of an active lifestyle and involvement of individuals in PA programmes [6,7]. Public health providers and policy makers can help their citizens achieve the recommended PA levels, promoting PA programmes among other actions [8,9], while ensuring optimal utilization of community resources. It is widely accepted that the benefits of such programmes depend on adherence to exercise, which is influenced by degree of enjoyment and satisfaction [10-15]. Moreover, one of the most important factors in customer satisfaction is quality of service [16-18]. Therefore, continual improvements in PA programmes for the elderly may play a significant role in elderly satisfaction and adherence to PA.

In Portugal, there are several PA programmes for elderly people developed by the local government, but very few are committed to their own assessment [19], which is a desirable prerequisite to continuous quality improvements [20,21]. Indeed, the *National Center for Chronic Disease Prevention’s Division of Nutrition and Physical Activity* described a set of recommendations and strategies to improve programmes, developing new approaches and highlighting the need for effective programme evaluation [22,23]. Likewise, World Health Organization (WHO) guidelines for the evaluation of health promotion emphasize the need to evaluate and propose the allocation of adequate evaluative resources [24].

With the purpose of helping PA programmes for the elderly to improve their quality, a Delphi process based on the criteria and sub-criteria from the European Foundation for Quality Management Excellence Model (EFQM) [18] and PA guidelines for older adults [3,25] was conducted, to identify practices that must be observed when assessing the quality of PA programmes for the elderly [26]. The study led to the creation of an instrument called Q-STEPS (Quality Self-assessment Tool for Exercise Programmes for Seniors).

Q-STEPS is a continuous improvement tool designed to be flexible and adaptable and consists of 165 statements that assess nine criteria involved in the implementation of PA programmes for the elderly: five criteria assess *Enablers* (Leadership, Policy & Strategy, People, Partnership & Resources, and Processes) and four criteria assess the *Results* (Customer results, People Results, Society Results, and Key Performance Results). The framework promotes and supports management teams to administer more efficiently and effectively, and get closer to meeting and exceeding customers’ needs [27].

For some authors [28-30], quality management processes and tools provide a practical way for organisations to identify and overcome the barriers to the improvement. Therefore, since this tool offers a framework tailored to evaluate PA programmes for the elderly, the information obtained through such evaluations would be useful for organizations seeking to improve the quality of their services, which may increase participation in PA. As mentioned previously, the Q-STEPS process consists of a self-assessment practice [27], which should encourage the development of an improvement action plan. Indeed, the international experience in different areas working within this type of frameworks, focused on a cycle of “plan-do-study-act” [31], has contributed significantly to system improvements [32-35]. Moreover, the Q-STEPS is not a one-off activity: it can involve continual self-assessment and later, external assessment, if that is in the interest of the organization.

Like other processes of conducting self-assessment [27,36], the Q-STEPS requires different *steps*, which will be explained ahead.

### Q-STEPS process steps

The starting point is to gain leadership commitment for using the Q-STEPS and then to plan the process. Subsequently, a team of staff representing different kinds of expertise within the programme’s structure is selected and trained to be responsible for managing the self-assessment process, mastering the tool. For this stage, the Q-STEPS Brochure (also available online) provides the information to increase the team’s awareness and understanding of the tool and the basic forms needed for the evaluation process: the Programme Characterization form (Q-SPC), the Checklist form (Q-SHK) and the Action Plan form (Q-SAP).

Briefly, 1) the Q-SPC is a general data collection sheet to be completed by the programmes wishing to enrol in a Q-STEPS process; 2) the Q-SHK is a list with the best practices (statements) included in the Q-STEPS instrument, which must be scored out of four, based upon the following:

1. *Poor*: there is little or no evidence of the specific practice, or no awareness or commitment to create or develop the practice.

2. *Fair*: there is evidence that the processes of planning and developing the practice has commenced and is progressing.

3. *Good*: there is evidence that demonstrates the practice is in place.

4. *Excellent*: the practice has been in place long enough to evidence the impact of what it has achieved in terms of real outcomes; and 3) the Q-SAP is a form that includes four major elements: the best practice statement that will be the target of an action; the specific tasks, including what will be done and by whom; the time horizon to achieve actions; and the resource allocation that are available for specific activities.

Each member of the self-assessment team completes the Q-SHK and the team collate performance information (e.g., customer survey results, employee surveys, programme budget, etc…). The filled checklist should be agreed on, and where possible, it would be relevant to discuss the issues with other employees and stakeholders, correctly identifying strengths and areas for improvement, showing priorities, responsibilities and goals for all actions. The sum of the scores in each sub-criterion/criteria is converted into averages and the percentages of achievements are calculated for each sub-criterion/criteria.

After recording the results and communicating them to stakeholders, the action plan is prepared and documented in the Q-SAP form previously mentioned. According to the action plan and the strategic directions, a responsible person should be pointed out and the appropriate resources to implement actions should be available. Finally, the whole self-assessment process should be subject to regular reviews, once per year.

When the self-assessment has been undertaken in accordance with these steps, the final goal should be the improvement, based on knowledge acquired from the self-assessment [36].

**Figure 1:**
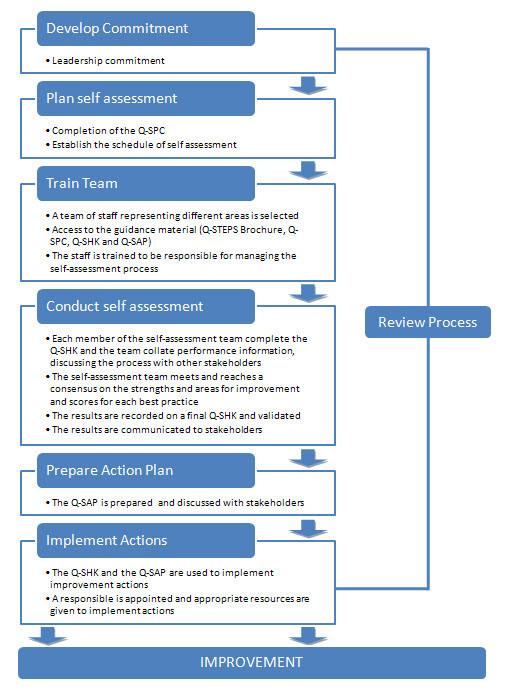
Q-STEPS process steps.

Q-STEPS process steps are illustrated in Figure 1.

### The aim and objectives

Despite the numerous PA programmes for the elderly that have been developed in recent years - especially by the Public Local Administration - their evaluation is scarce [19]. Moreover, the Q-STEPS has been created with the aim of identifying practices that must be observed when assessing the quality of PA programmes for the elderly [26] and gathering information that would be useful for organizations seeking to improve their services. Since a feasibility study has never been conducted to assess whether the tool would work in a practice setting and considering it as a key-point for the judgement of the viability of this tool, it seems of importance to accomplish it. In this context, the primary purpose of this study is 1) to determine feasibility, acceptability and usability of the Q-STEPS. Secondary purposes of the study are: 2) to examine the quality of the PA programmes for elderly people developed by the Portuguese Local Administration over a three-year period of self-assessments in terms of: a) Enabler domains (Leadership, Policy and Strategy, People, Partnership and Resources, Processes); b) Result domains (Customer Results, People Results, Society Results and Key Performance Results); and 3) to estimate the association between the use of Q-STEPS and some indicators relating to the elderly participants, during the three self-assessments, such as: attendance rates, physical fitness, health-related quality of life and the elderly’s perceived quality of the programme.

## Methods/design

**Figure 2:**
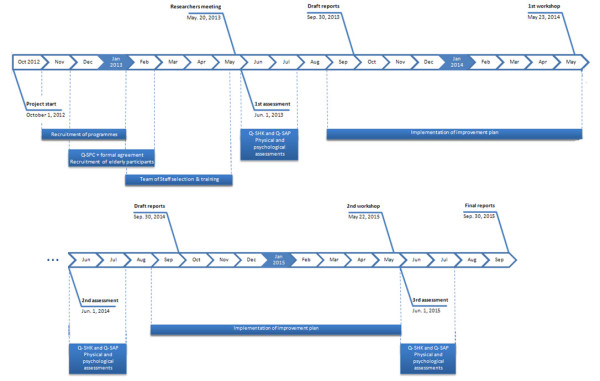
Q-STEPS study schedule.

The study will adopt the following methodology, as defined below on the viewable schedule (Figure 2).

### Recruitment of participating PA programmes

The study will be conducted in PA programmes for elderly adults from mainland Portuguese municipalities. According to the results of a previous study [37], the sample size is estimated to be between 20 and 30 PA programmes which represents a considerable portion (between 16% and 24%) of the population size. In order to combine differences found in several existing programmes [37], inclusion criteria for the sample implied that at least one of the following conditions should be verified: i) programmes should belong to a District Capital in order to apply a geographic criterion; ii) programmes should include the following cumulative criteria: a) must have been in practice for 10 years or more [38], b) must have had two or more different types of activities [39,40], and c) must have had a frequency of two or more times a week [3].

The invitation for participation will be sent electronically to the mainland Portuguese municipalities, and a comprehensive explanation of the purpose and study design will be completed. Two reminders will be sent to those who have not responded within two weeks. An investigator’s contact details will be provided for participants to raise questions or doubts about the study. After expressing interest in participating, a formal agreement will be negotiated with each PA programme, which should be aware of their engagement during three annual cycles of self-assessment, feedback, planning and implementation. The expectations and the counterparts of both parties will be explicit in this document. Among other requirements, it will always be a part of the commitment to providing the data of elderly’s attendance rates. Moreover, all elderly participants from this PA programmes will be asked to volunteer for this study, completing physical and psychological assessments, which will be described further on in this paper. After the recruitment period, volunteers will be invited to a preliminary meeting in which they will be informed about the nature, risks and procedures of the study. A written informed consent will be request from those who agree to participate, consistent with the principles of the Helsinki Declaration. The Q-SPC will be filled.

### Self-assessment team selection and training

A staff member from each participating PA programme will be designated as responsible for running the Q-STEPS process, ensuring that all necessary information and documentation is provided to the self-assessment team, supporting contacts and information distribution within the remaining members. The designated member (hereinafter referred to as the “facilitator”) will have training and support provided by research project staff. Training for facilitators consisted of a detailed manual and a 2-day course where the Q-STEPS should be introduced and the purposes and nature of the self-assessment procedure explained. During this process, facilitators will also be accomplished for the remaining assessments, as is the case of the physical and psychological assessments of elderly participants.

The self-assessment team should include members from different sectors/functions.

An online platform will fully support the training of the self-assessment team, providing consistent guidance for assessing.

### The annual learning cycle

The process will adopt a participative quality improvement approach that features annual learning cycles of:

1) self-assessment with the Q-STEPS;

2) feedback to and interpretation of results involving programme’s staff;

3) action planning to achieve changes in PA programmes;

4) implementation of strategies/actions for change; and

5) review process through further self-assessment.

In each of these steps, which have been previously described (please see: Q-STEPS exercise section), members of the research team will make periodic visits to every programmme in order to become aware of the process and clarify any doubts that may exist.

### The workshop

Data from the self-assessments will be analysed by the research team. Feedback of results to the PA programme self-assessment teams will be conducted in a workshop, held annually. During each workshop, each member of the self-assessment team will be given a questionnaire in order to explore barriers/enhancers to full participation in the Q-STEPS process. The questionnaire is based on the key process assessment criteria proposed by Platts [41,42]: a) feasibility – can the process be followed?; b) usability – is the tool easy to use?; and c) utility – is the process worth following? Each criterion will be divided into several sub-criteria: *feasibility* – availability of information, timing and participants; *usability* – clarity, ease of use and appropriateness; and *utility* – relevance, usefulness, facilitation and confidence. The questionnaire design will use a four point Likert scale (1 = strongly agree; 2 = agree; 3 = disagree; 4 = strongly disagree). In addition to the rating criteria, a graphic rating scale will be used to measure the degree of confidence on the process (0%-100%). Open-ended questions to capture relevant issues about the Q-STEPS process and suggestions for improvement will also be part of the questionnaire.

The intention of the workshop is to monitor the process of self-assessment and disseminate the results by all stakeholders [43]. Programme staff will be encouraged to play an active role in the workshop, exploring and sharing lessons and best practices between participating programmes.

### Data analysis

The study will collect a range of process and outcome data [23] that will be used to examine the research aims. These include:

1.) The determination of feasibility, acceptability and usability of the Q-STEPS through analysis of data from the questionnaire to members of the self-assessment teams, carried out during the workshops.

2.) The examination of the quality of the PA programmes for elderly people developed by the Portuguese Local Administration over a three-year period, through the results of self-assessments scores.

3.) The examination of associations between the use of the Q-STEPS and some indicators relating to the elderly participants, using outcomes from different assessments. Data will be collected at three points in time, coinciding with the self-assessment with the Q-STEPS.

3.1) The Senior Fitness Test (SFT) will be used to assess physical fitness [44]. This battery consists of six assessment items, designed and validated to assess the physiological parameters that support physical functionality and mobility in older adults. The test items include lower body strength (30-s chair stand), upper body strength (30-s arm curl), aerobic endurance (6-min walk test), lower body flexibility (chair sit-and-reach), and dynamic balance and agility (the 8-ft up-and-go). During assessments, the test administrator and the time of day used for collection will remain constant.

3.2) The health-related quality of life will be assessed by the Portuguese version of the Medical Outcomes Study Short-Form Health Survey (MOS SF-36), a standard generic international instrument to assess functional health and well-being from the participant’s point of view, including 36 items and covering eight dimensions: physical functioning (PF; ten items), role limitations due to physical problems (RP; four items), bodily pain (BP; two items), general health (GH; five items), vitality (VT; four items), social functioning (SF; two items), role limitations due to emotional problems (RE; three items), and mental health (MH; five items) [45-47]. There is also a single separate item that is used to assess any change in health from the previous year. The SF-36 will be administered by interview, and scores will be calculated using the methods set out by Ware and collaborators [47]. The scores range from 0 to 100, with higher scores indicating better functional health and well-being.

3.3) The elderly’s perceived quality of programmes will be measured using the QUESPMAFI, an instrument adapted and validated for the Portuguese population [48].

3.4) Administrative data relating to attendance of the elderly will be reported by each programme. Attendance at programmes will be accounted as the means ± standard deviations of attended sessions relative to the total number of possible sessions [49].

The Programme Characterization form (Q-SPC) will provide the following information that will be analysed and compared with the other variables described above: geographic localization, name and objectives of PA programmes, age of the PA programme, number of participants, characteristics of age groups and participants’ average age, number of employees, number of activities offered in the PA programme, frequency of the programme (days/week), number of sports facilities, programme fees, quality initiatives previously developed by the programme, name of the organization that delivers the programme, and identification details of the PA programme’s coordinator (name, gender, age, qualification and contact).

#### Statistics

Descriptive statistics will be used to characterize all the sample variables. Data will be tested for normality, homogeneity of variance and independence. Contingency tables will test possible associations between variables with *χ*^2^ test or, in the case of small-expected frequencies, Fisher’s exact test. Measures of self-assessment results and outcomes will be compared using *t*-test or *χ*^2^ test, for continuous variables or for nominal variables respectively (or the non-parametric analogue, the Mann–Whitney test, if data does not meet the assumptions of parametricity). The Pearson’s Correlation coefficient will be used to test the hypothesis of independence of variables (or the non-parametric analogue, the Spearman rank order correlation coefficient). Repeated measures will be examined using repeated measures ANOVA (or the non-parametric analogue, the Friedman’s test).

All analysis will be performed with the Statistical Package IBM-SPSS Statistics, version 19.0 or superior. The level of significance will be set at p < 0.05.

Reports will contain results from statistical analyses.

## Ethics

The study was approved by the Institutional Review Board of the Faculty of Sport - University of Porto and the Portuguese Foundation for Science and Technology (reference: SFRH/BD/36796/2007).

## Conclusion

Ongoing monitoring and evaluation of PA programmes for the elderly is needed to provide sound empirical evidence of what makes a programme sustainable and effective. The information obtained through such evaluations would be useful for organizations seeking to improve their services and would help them guide interventions toward excellence. This may also help to inform policy makers and other community services, in order to adopt quality criteria in their actions.

This study will help us to understand if the Q-STEPS can be used in programme evaluation, with viability. This study will also provide an opportunity to assess, over time, the quality of PA programmes for elderly people developed by the Portuguese Local Administration, using a new tool created for this purpose, as well as comparing the results of this assessment with other data/outcomes related to programmes.

The Q-STEPS study will contribute directly to the evidence based on effectiveness of continuous quality improvement approaches, in order to improve customer satisfaction and adherence to PA programmes targeting the ageing population. This comprehensive evaluation will also add significant new knowledge regarding the characteristics associated with a sustainable public service.

## Competing interests

The authors declare that they have no competing interests.

## Authors’ contributions

AIM conceived the project and its design and participated in drafting and editing the manuscript. MJR and MA contributed to the development of the study, participated in its design and coordination. PS, AOT and RS assisted in the development of the study and revision of the manuscript. JM and JC participated in the coordination of the study, contributed to the procurement of the funding and were involved in revising the manuscript critically. All authors read and approved the final manuscript.
